# Acceptability and retention of the key population‐led HIV treatment service for men who have sex with men and transgender women living with HIV in Thailand

**DOI:** 10.1002/jia2.26062

**Published:** 2023-02-09

**Authors:** Sita Lujintanon, Sorawit Amatavete, Prattana Leenasirimakul, Jantana Meechure, Preudtipong Noopetch, Supakarn Sangtong, Satayu Sittikarn, Poonnanat Phoopisutthisak, Pich Seekaew, Stephen Mills, Praphan Phanuphak, Reshmie A. Ramautarsing, Nittaya Phanuphak

**Affiliations:** ^1^ Department of Epidemiology Johns Hopkins Bloomberg School of Public Health Baltimore Maryland USA; ^2^ Institute of HIV Research and Innovation Bangkok Thailand; ^3^ Nakornping Hospital Chiang Mai Thailand; ^4^ Hangdong Hospital Chiang Mai Thailand; ^5^ Hatyai Hospital Songkhla Thailand; ^6^ Mplus Foundation Chiang Mai Thailand; ^7^ CAREMAT Chiang Mai Thailand; ^8^ Rainbow Sky Association of Thailand Bangkok Thailand; ^9^ Department of Epidemiology Columbia University Mailman School of Public Health New York New York USA; ^10^ FHI 360 and LINKAGES Bangkok Thailand

**Keywords:** HIV, antiretroviral therapy, differentiated service delivery, men who have sex with men, transgender women, Thailand

## Abstract

**Introduction:**

In Thailand, where the HIV epidemic is concentrated among key populations (KPs), particularly men who have sex with men (MSM) and transgender women (TGW), an HIV service delivery model tailored to KPs was piloted. This study evaluated the acceptability and retention of clients who accepted and declined the KP‐led HIV treatment service.

**Methods:**

A retrospective cohort study was conducted using secondary data from three community‐based organizations (CBOs) and three hospitals in Thailand. KP lay providers were trained to lead HIV treatment service in which MSM and TGW living with HIV received counselling and a 3‐month antiretroviral therapy (ART) supply at CBOs. Thai MSM and TGW who were at least 18 years, on ART for at least 6–12 months, without co‐morbidities/co‐infections, and virally suppressed were eligible and offered the service. Those who declined received ART via other service models offered by the hospitals and served as a comparison group.

**Results:**

Of 220 clients screened between February 2019 and February 2020, 72% (159/220) were eligible of which 146 were MSM and 13 were TGW. Overall, 45% (72/159) accepted the KP‐led service. Of those who declined, 98% (85/87) preferred to see the physician at the hospital. After 12 months of follow‐up, among those accepted, 57% were in care at the CBO, 32% were referred back to and in care in other service models offered by the hospital, 10% were successfully transferred out to other hospital and 1% were lost to follow‐up (LTFU); among those declined, 92% were in care in any service models offered by the hospital, 5% were successfully transferred out to other hospital, 2% were LTFU and 1% died (*p*‐value<0.001).

**Conclusions:**

Despite moderate acceptability and retention in care at the CBO among the clients accepting the KP‐led service, almost all clients were engaged in care overall. Multiple service models that meet the preferences and needs of KPs living with HIV should be available to optimize engagement in care.

## 1 INTRODUCTION

Globally, men who have sex with men (MSM) and transgender women (TGW) have a 28‐ and 14‐times greater risk of acquiring HIV, respectively, when compared to the general population [1]. In Thailand, the overall number of new HIV infections has been on the decline with an estimated number of new infections of 5585 in 2022 [2]. However, key populations (KPs), specifically MSM and TGW, were accounted for approximately 50% of new infections [2]. While antiretroviral therapy (ART) is free of charge through the national health insurance in Thailand, only 31.8% of MSM with HIV and 6.0% of TGW with HIV were estimated to be on ART; only 66.7% and 44.3% of those on ART were virally suppressed, respectively [3]. The suboptimal service uptake might be due to stigma and discrimination in the healthcare setting, which caused many MSM and TGW to delay or avoid seeking care [2, 4]. Despite the national HIV guidelines having recommended task shifting, where certain health tasks are being redistributed among workforces and communities, since 2017 [5], Thailand's HIV care system remains largely centralized at secondary and tertiary care facilities [6]. Consequently, ART clients may experience long visits, inflexible schedules and clinic overcrowding [6].

The benefits of community‐based ART delivery on retention and viral suppression have been demonstrated through programme implementation and research in sub‐Saharan Africa; however, most studies were conducted on the general population [7, 8, 9, 10]. In the HIV epidemic concentrated on KPs, differentiated service delivery through decentralization and task shifting are needed to improve service uptake and health outcomes [11]. In Thailand, the Key Population‐Led Health Service (KPLHS) model has allowed the trained KP lay providers at community‐based organizations (CBOs) to lead the provision of health services that are needed in their communities [12, 13, 14]. This model has been replicated and described in other countries in Southeast Asia [15, 16, 17, 18]. While the model initially focused on HIV testing [19], linkage to care [20] and prevention [21, 22], here, we piloted a KP‐led HIV treatment service for people established on ART.

This manuscript evaluates the acceptability and retention of the KP‐led HIV treatment service for MSM and TGW living with HIV in Thailand.

## 2 METHODS

### 2.1 Study design and participants

This is a retrospective cohort study using existing medical records from CBOs and hospitals. The KP‐led HIV treatment service was implemented at three CBOs: Mplus Chiang Mai and Caremat in Chiang Mai, and RSAT Hatyai in Songkhla, in collaboration with three hospitals: Nakornping Hospital (since February 2019) and Hangdong Hospital (since December 2019) in Chiang Mai, and Hatyai Hospital (May 2019) in Songkhla. The CBOs provided HIV/sexually transmitted infection (STI) testing and counselling, and pre‐exposure prophylaxis and post‐exposure prophylaxis distribution to their clients who were mainly MSM and TGW. Nakornping Hospital and Hatyai Hospital were public tertiary hospitals; Hangdong Hospital was a medium‐sized public community hospital. Throughout the implementation of the KP‐led service, technical support was provided by the Institute of HIV Research and Innovation (IHRI).

The eligibility criteria for the KP‐led service included being MSM and TGW living with HIV who were at least 18 years, Thai citizens, on ART for at least 6–12 months, having no co‐morbidities and co‐infections, and having achieved viral suppression at least once. This analysis included people who were offered the KP‐led service between February 2019 and February 2020. Those who were offered but declined the KP‐led service and continued to receive ART via other service models offered by the hospitals served as a comparison group.

### 2.2 Intervention design and preparation

The KP‐led HIV treatment service was supported by an ongoing and long‐term, multi‐stakeholder strategy bundle to integrate KPLHS into the national health system [14]. The standard operating procedure (SOP) was designed by CBO, partnered hospital and IHRI through a series of feedback meetings and SOP document revisions. This resulted in a slightly different service flow for each of the CBO–hospital partnership. Before the service initiation, the KP lay providers were trained by the partnered hospitals and IHRI, including didactic and practical training on counselling, HIV/STI testing and prevention, and ART distribution. The lay providers had to pass the test to be certified by IHRI in order to provide any services at the CBOs [12, 14]. SOP training and service dry run led by the partnered hospitals and IHRI were conducted before the service initiation.

### 2.3 KP‐led HIV treatment service procedures

**Figure 1:**
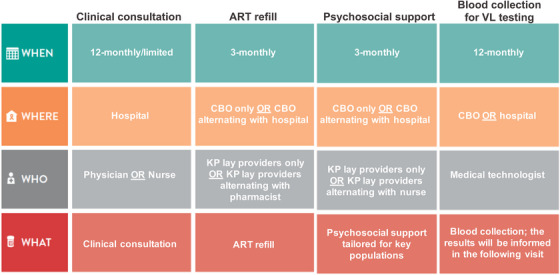
Differentiated service delivery building blocks of the key population‐led community‐based HIV treatment service. Abbreviations: ART, antiretroviral therapy; CBO, community‐based organization; KP, key population; VL, viral load.

The service is summarized in Figure 1. The service was led by the trained KP lay providers with the support of the HIV clinic team from the partnered hospitals. MSM and TGW living with HIV who were receiving ART at the hospitals according to their national health insurance coverage were screened according to the eligibility criteria by the HIV clinic staff. Eligible clients were informed and offered the KP‐led service by the HIV clinic or CBO staff. Upon accepting the service, the clients were scheduled to refill ART at the CBO in the following visit. Those who declined continue to refill ART via other service models offered by the hospitals as shown in Table 1.

**Table 1:** Routine ART refill service models provided by the partnered hospitals

Service models	Description	Hospitals provided
Conventional service	ART refill at the hospital after receiving clinical consultation from a physician.	All hospitals
After‐hours clinic service	ART refill at the hospital after receiving clinical consultation from a physician during evening or weekend hours with a fee.	Nakornping Hospital, Hatyai Hospital
Fast‐track refill service	ART refill at the hospital's pharmacy without seeing a clinical provider.	Nakornping Hospital
Nurse‐led clinical consultation service	ART refill at the hospital after receiving clinical consultation from a nurse.	Hatyai Hospital, Hangdong Hospital
Mailing service	ART delivery to home via postal service with a fee.	All hospitals

Abbreviation: ART, antiretroviral therapy.

Before the visit, a physician and/or a nurse at the hospital reviewed the medical records of the clients who were due for ART refill at the CBO, prescribed the ART if clinically eligible and schedule a phlebotomy if due. The pharmacist prepared the ART package, including the 3‐month ART supply and an appointment card for the next visit, and the CBO staff transported the ART package to the CBO.

On the day of the ART refill visit at the CBO, the KP lay provider provided adherence and psychosocial support counselling, and dispensed the ART supply to the clients. The clients had to return to the hospital for phlebotomy on a separate visit for the annual viral load (VL) testing and routine lab work (i.e. complete blood count, fasting blood sugar, creatinine, total cholesterol, triglyceride, alanine transaminase, hepatitis B surface antigen, hepatitis C antibody, syphilis [Venereal Disease Research Laboratory] and CD4 cell count according to the national guidelines), except for the clients at RSAT Hatyai where phlebotomy could be conducted at the CBO and the samples were processed at Hatyai Hospital. Phlebotomy and ART refill visits were combined at Hangdong Hospital. VL and other lab results would be informed in the following visit. Clients with clinical concerns would be referred to see a physician and/or nurse at the hospital for further investigation. Clients could switch to maintain their ART via other service models offered by the hospitals at any time they wished. This service had no fee, and the cost of ART and routine lab tests was covered by national health insurance.

The CBOs, hospitals and IHRI conducted quality assurance/quality improvement every quarter or as necessary by reviewing the medical record forms, holding feedback meetings and presenting service performance data.

### 2.4 Outcome measures

The primary outcomes were acceptability and retention. Acceptability was defined as agreeing to receive the KP‐led service among the clients who were screened and offered the service. Retention was defined as being in care and receiving ART at the CBO and/or the hospital at months 3, 6, 9 and 12, and categorized as in care, referred back to the hospital (defined as clients who received the KP‐led service in the previous visit and were in care at the hospital in the following visit), transferred out to other hospital, loss to follow‐up (LTFU, defined as clients who were unable to be contacted by the HIV clinic or CBO staff and had no medical records of receiving ART for more than 45 days) and death. Follow‐up data collection stopped upon the transfer for clients who successfully transferred out to receive care at other hospital. Secondary outcomes were VL testing and viral suppression, which was defined as HIV‐1 RNA <50 copies/ml. Age and duration on ART variables were also included in the analysis.

### 2.5 Statistical analysis

Descriptive statistics were used to summarize the outcomes and presented as percentages for categorical variables and as median and interquartile range (IQR) for continuous variables. The proportions of clients accepted and retained at each follow‐up time point were assessed. Two analyses were conducted for retention. The primary analysis described the retention status of all clients at each time point. The secondary analysis excluded the clients who transferred out to other hospital from the denominator as they were deemed to be in care but we were unable to collect their data. Since our study combined the data from three different sites and the follow‐up time overlapped with the COVID‐19 pandemic, which might cause a change in clients’ model preference and/or circumstance, descriptive analyses stratified by hospitals and time of the first ART refill visit after screening were conducted to explore any heterogeneity in client characteristics and outcomes.

Statistical analysis was conducted with R version 4.1.2 (R Foundation for Statistical Computing, Vienna, Austria)/RStudio version 1.3.1093 (RStudio PBC, Boston, MA, United States).

### 2.6 Ethical consideration

HIV treatment was provided according to national standards. The informed consent process was waived as the secondary data from the CBO and hospital databases were collected without personal identifiers. The study was approved by the Central Research Ethics Committee (IRB: 009/62SCm; NCT: 04383769).

## 3 RESULTS

**Figure 2:**
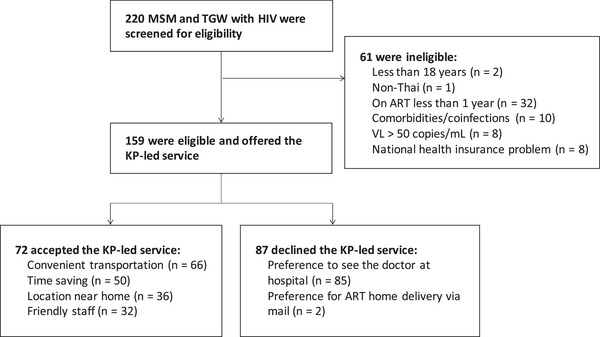
Overall screening cascade for the key population‐led community‐based HIV treatment service. Abbreviations: ART, antiretroviral therapy; KP, key population; ml, millilitre; MSM, men who have sex with men; TGW, transgender women; VL, viral load.

A total of 220 MSM and TGW with HIV were screened. Of those, 72% (159/220) were eligible for the KP‐led HIV treatment service (Figure 2). Some reasons for being ineligible were being on ART less than 1 year (53%, 32/61) and had co‐morbidities/co‐infections (16%, 10/61). The overall acceptability was 45% (72/159). The reasons for accepting included convenient transportation (92%, 66/72), time‐saving (69%, 50/72), location near home (50%, 36/72) and friendly staff (44%, 32/72). The reasons for declining the service among eligible clients were preference to see the physician at the hospital (98%, 85/87) and preference to have ART delivered to their home via mail (2%, 2/87). Instead of receiving the KP‐led service, these clients received the conventional (85%, 74/87), after‐hours clinic (10%, 9/87), mailing (3%, 3/87) and nurse‐led services (1%, 1/87).

Of all eligible clients, 92% (146/159) were MSM; only 13 clients were TGW (Table 2). The overall median age (IQR) among accepted and declined clients was 29.0 (25.0–35.0) and 30.0 (26.0–34.0) (*p*‐value = 0.460), respectively. The overall median (IQR) duration on ART of those accepted and declined was 3.75 years (2.20–4.33) and 3.64 years (2.41–5.08) (*p*‐value = 0.387), respectively.

**Table 2:** Characteristics of men who have sex with men and transgender women who were offered the key population‐led community‐based HIV treatment service

	MSM			TGW			Overall		
	Accepted	Declined		Accepted	Declined		Accepted	Declined	
	(*N* = 64)	(*N* = 82)	*p*‐value	(*N* = 8)	(*N* = 5)	*p*‐value	(*N* = 72)	(*N* = 87)	*p*‐value
Age (years)									
Median (IQR)	29.0 (25.8–34.3)	29.5 (26.3–34.0)	0.568^a^	28.5 (24.5–35.0)	34.0 (26.0–38.0)	0.509^a^	29.0 (25.0–35.0)	30.0 (26.0–34.0)	0.460^a^
Age (years)			0.268^b^			1.000^c^			0.240^b^
<25	13 (20%)	10 (12%)		2 (25%)	1 (20%)		15 (21%)	11 (13%)	
> = 25	51 (80%)	72 (88%)		6 (75%)	4 (80%)		57 (79%)	76 (87%)	
Duration on ART (years)									0.387^a^
Median (IQR)	3.78 (2.61–4.34)	3.49 (2.48–4.96)	0.718^a^	2.21 (1.74–3.95)	3.98 (2.21–5.68)	0.306^a^	3.75 (2.20–4.33)	3.64 (2.41–5.08)	
Duration on ART (years)			0.059^b^			0.524^c^			0.034^b^
1	10 (16%)	12 (15%)		3 (38%)	1 (20%)		13 (18%)	13 (15%)	
2	11 (17%)	25 (30%)		2 (25%)	1 (20%)		13 (18%)	26 (30%)	
3	15 (23%)	11 (13%)		1 (12%)	1 (20%)		16 (22%)	12 (14%)	
4	19 (30%)	14 (17%)		2 (25%)	0 (0%)		21 (29%)	14 (16%)	
5 and above	9 (14%)	20 (24%)		0 (0%)	2 (40%)		9 (12%)	22 (25%)	
Partner hospital			<0.001^c^			0.767^c^			<0.001^c^
Nakornping Hospital	18 (28%)	3 (4%)		3 (38%)	2 (40%)		21 (29%)	5 (6%)	
Hangdong Hospital	11 (17%)	2 (2%)		2 (25%)	0 (0%)		13 (18%)	2 (2%)	
Hatyai Hospital	35 (55%)	77 (94%)		3 (38%)	3 (60%)		38 (53%)	80 (92%)	

Abbreviations: ART, antiretroviral therapy; IQR, interquartile range; MSM, men who have sex with men; TGW, transgender women.

^a^Kruskal–Wallis test.

^b^Chi‐squared test.

^c^Fisher's exact test.

**Figure 3:**
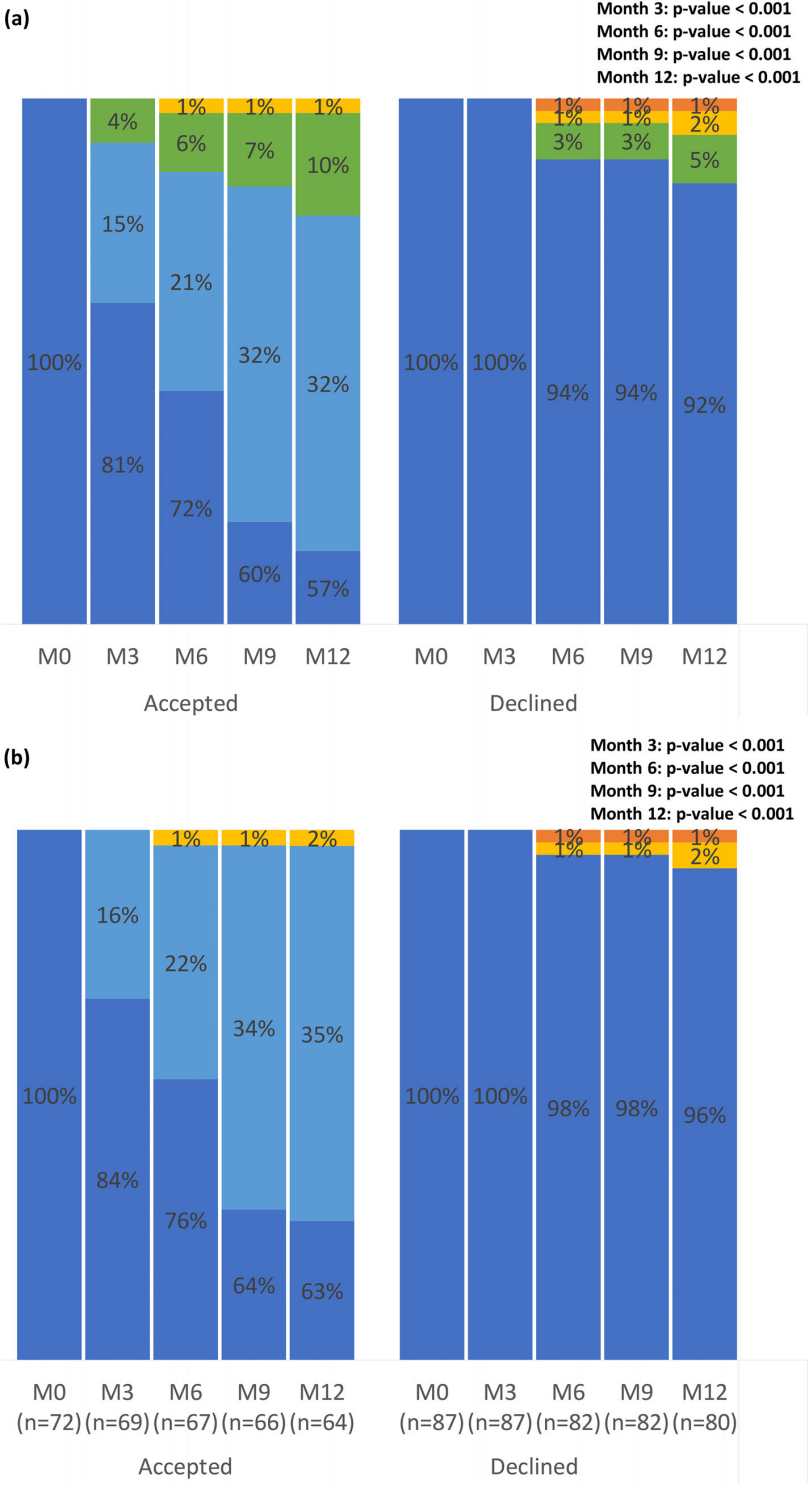
Retention at months 3, 6, 9 and 12: (a) including those who transferred out in the denominator and (b) excluding those who transferred out from the denominator. Abbreviations: CBO, community‐based organization; LTFU, loss to follow‐up; M, month.

Among those who accepted the KP‐led service, 57% (41/72) were in care at the CBO, 32% (23/72) were referred back to and in care in other service models offered by the hospital, 10% (7/72) were successfully transferred out to other hospital and 1% (1/72) were LTFU by month 12 (Table 3 and Figure 3a). Those who were referred back to the hospital at month 12 received conventional (39%, 9/23), mailing (30%, 7/23), nurse‐led clinical consultation (26%, 6/23) and fast‐track refill services (4%, 1/23). Among those who declined the KP‐led service, 92% (80/87) were in care in any models, 5% (4/87) were successfully transferred out to other hospital, 2% (2/87) were LTFU and 1% (1/87) died by month 12 (Table 3 and Figure 3a). When excluding those who transferred out, 63% and 35% of the accepted clients were in care at the CBO and in other service models offered by the hospital, respectively; 96% of the declined clients were in care in any service models at month 12 (Figure 3b). The overall retention patterns of clients who accepted and declined the KP‐led service were statistically different (*p*‐value<0.001).

**Table 3:** Retention, viral load testing and viral suppression outcomes stratified by populations

	MSM			TGW			Overall		
	Accepted	Declined		Accepted	Declined		Accepted	Declined	
	(*N* = 64)	(*N* = 82)	*p*‐value	(*N* = 8)	(*N* = 5)	*p*‐value	(*N* = 72)	(*N* = 87)	*p*‐value
Retention at month 3			<0.001^a^			1.000^a^			<0.001^a^
In care	51 (80%)	82 (100%)		7 (88%)	5 (100%)		58 (81%)	87 (100%)	
Referred back to hospital (CBO arm only)	10 (16%)	NA		1 (12%)	NA		11 (15%)	NA	
Transferred out to other hospital	3 (5%)	0 (0%)		0 (0%)	0 (0%)		3 (4%)	0 (0%)	
LTFU	0 (0%)	0 (0%)		0 (0%)	0 (0%)		0 (0%)	0 (0%)	
Death	0 (0%)	0 (0%)		0 (0%)	0 (0%)		0 (0%)	0 (0%)	
Retention at month 6			<0.001^a^			0.487^a^			<0.001^a^
In care	46 (72%)	77 (94%)		6 (75%)	5 (100%)		52 (72%)	82 (94%)	
Referred back to hospital (CBO arm only)	13 (20%)	NA		2 (25%)	NA		15 (21%)	NA	
Transferred out to other hospital	4 (6%)	3 (4%)		0 (0%)	0 (0%)		4 (6%)	3 (3%)	
LTFU	1 (2%)	1 (1%)		0 (0%)	0 (0%)		1 (1%)	1 (1%)	
Death	0 (0%)	1 (1%)		0 (0%)	0 (0%)		0 (0%)	1 (1%)	
Retention at month 9			<0.001^a^			0.075^a^			<0.001^a^
In care	40 (62%)	77 (94%)		3 (38%)	5 (100%)		43 (60%)	82 (94%)	
Referred back to hospital (CBO arm only)	18 (28%)	NA		5 (62%)	NA		23 (32%)	NA	
Transferred out to other hospital	5 (8%)	3 (4%)		0 (0%)	0 (0%)		5 (7%)	3 (3%)	
LTFU	1 (2%)	1 (1%)		0 (0%)	0 (0%)		1 (1%)	1 (1%)	
Death	0 (0%)	1 (1%)		0 (0%)	0 (0%)		0 (0%)	1 (1%)	
Retention at month 12			<0.001^a^			0.231^a^			<0.001^a^
In care	36 (56%)	75 (91%)		5 (62%)	5 (100%)		41 (57%)	80 (92%)	
Referred back to hospital (CBO arm only)	20 (31%)	NA		3 (38%)	NA		23 (32%)	NA	
Transferred out to other hospital	7 (11%)	4 (5%)		0 (0%)	0 (0%)		7 (10%)	4 (5%)	
LTFU	1 (2%)	2 (2%)		0 (0%)	0 (0%)		1 (1%)	2 (2%)	
Death	0 (0%)	1 (1%)		0 (0%)	0 (0%)		0 (0%)	1 (1%)	
Viral load testing in the past 12 months		0.049^b^			0.293^a^			0.023^b^	
Tested	41 (64%)	38 (46%)		6 (75%)	2 (40%)		47 (65%)	40 (46%)	
Not tested	23 (36%)	44 (54%)		2 (25%)	3 (60%)		25 (35%)	47 (54%)	
Viral load results in the past 12 months		0.030^a^			0.293^a^			0.016^a^	
<50 copies/ml	41 (64%)	37 (45%)		6 (75%)	2 (40%)		47 (65%)	39 (45%)	
≥50 copies/ml	0 (0%)	1 (1%)		0 (0%)	0 (0%)		0 (0%)	1 (1%)	
Not tested	23 (35.9%)	44 (53.7%)		2 (25.0%)	3 (60.0%)		25 (34.7%)	47 (54.0%)	

Abbreviations: CBO, community‐based organization; LTFU, loss to follow‐up; ml, millilitre; MSM, men who have sex with men; NA, not applicable; TGW, transgender women; VL, viral load.

^a^Fisher's exact test.

^b^Chi‐squared test.

Of all accepted and declined clients, 65% (47/72) and 46% (40/87) received VL testing through the partnered hospitals (*p*‐value = 0.023). Furthermore, 65% (47/72) of the accepted clients were virally suppressed in the past 12 months versus 45% (39/87) of the declined clients (*p*‐value = 0.016). All tested clients maintained viral suppression, except for one declined client who had a detectable VL.

The analyses stratified by hospitals and time of the first ART refill visit after screening are presented in the Supplementary Material (Tables S1 and S2). Heterogeneity by hospitals existed, including Chiang Mai sites had more younger people and Hangdong Hospital had more people recently been on ART (< 3 years). The acceptability among Hatyai Hospital clients was 32% (38/118), while the acceptability among Nakornping Hospital and Hangdong Hospital clients was 81% (21/26) and 87% (13/15), respectively. More clients from Nakornping Hospital (71%) were in care at the CBO by month 12; however, none of the accepted clients from Hangdong Hospital were in care at the CBO at month 12. VL testing coverage was high among accepted clients from Hatyai Hospital (92%) and Hangdong Hospital (77%); only 10% of clients from Nakorngping Hospital received VL testing. The acceptability increased from 22% before to 43% after 22 October 2019. At month 12, more clients who started refilling ART before this median date were retained in care at the CBO (68%) when compared to clients who started refilling ART at the CBO after (46%). Eight in the before and 15 in the after periods were referred back to the hospital (conventional: 4 vs. 5; fast‐track refill: 1 vs. 0; nurse‐led clinical consultation: 0 vs. 6; mailing: 3 vs. 4).

## 4 DISCUSSION

Our study demonstrated that the KP‐led service served as another HIV treatment option for MSM and TGW with HIV in addition to the services offered by the partnered hospitals. The acceptability (45%) was moderate and overall the accepted clients remained engaged in care either at the CBO (63%) or the hospital (35%) with only one LTFU and no death within a 12‐month follow‐up period. In addition, there was better VL testing coverage among clients who accepted the KP‐led service with all tested clients maintaining viral suppression. This service leveraged the ongoing efforts to integrate KPLHS into the national health system through long‐term multi‐stakeholder partnership, sustainable financing, capacity building and community leadership [14]. This study added to the growing body of evidence that the KPLHS approach can be effective for HIV treatment.

Almost half of our clients found the KP‐led service acceptable. This real‐world acceptability proportion was lower than our previous finding, whereby 62% of MSM and TGW who had been on ART for at least 1 year responded in a questionnaire that they preferred ART maintenance service to take place at the CBO [23]. Most of our clients accepted the KP‐led service because it eliminated the logistical barriers to ART that many people living with HIV broadly experienced [24, 25, 26]: convenient transportation (92%), time‐saving (69%) and location near home (50%). Having friendly staff (44%) might overcome the gender‐based stigma that some MSM and TGW experience in the conventional healthcare setting [2, 4, 27, 28, 29, 30]; however, reasons related to overcoming HIV‐based stigma were not mentioned. Unintended disclosure of HIV status is a common fear in other community‐based settings [24, 31], and some of our clients might decline the KP‐led service because they might not feel comfortable refilling ART at the CBO where their peers come for HIV testing and prevention services. Nonetheless, our KP‐led service was offered alongside with other HIV treatment service options provided by the partnered hospitals, which provided alternative and suitable choices for the declined clients. This demonstrated that one size does not fit all, even among the KPs, and various service options should be available for KP clients. Other service delivery modalities tailored to KPs, such as through telemedicine/ART mailing in which many of our clients preferred, should be explored [16].

Although some accepted clients switched to receive HIV treatment via other service models provided by the hospitals, almost all clients remained engaged in care. While 63% retention at the CBO was moderate, a similar retention proportion (60%) was reported among MSM after 6 months of receiving the community health worker‐led ART service in Nigeria [32]. Due to the limited number of studies on the KP‐led ART maintenance service, we compared our results to retention outcomes of other community‐based ART initiation services for MSM newly started on ART: our retention results were similar to a community‐based but physician‐led ART service in Nigeria (66% at month 6) [33] but lower than the results from a KP‐led test‐and‐treat service in the Philippines (76% at month 12) [17]. Moreover, 35% of the accepted clients were referred back to the hospital and were still engaged in care. This service utilization pattern in which the clients engaged in care by switching between available service models suggested that one KP‐led service model is not the solution. We need an HIV care system that has multiple service models available that are acceptable to KP clients in order to allow the provision of uninterrupted, people‐centred care as the clients transition between various stages of life, clinical stability and preferences or model availability [34]. This HIV care system could benefit from having an established referral system and clear communication between clients and providers as well as between providers and providers to ensure a smooth transition between service models.

Outcome heterogeneity between implementing sites existed. The lower acceptability at the Songkhla site might be explained by the older age and longer duration on ART of clients who might prefer to see physicians at the hospital. In addition, RSAT Hatyai was located <1 km from Hatyai Hospital and might not eliminate the logistical barriers related to location that some clients faced. Moreover, the outcomes stratified by the time of the first ART refill visit after screening were likely attributed by the later service initiation at Hangdong Hospital rather than the emergence of the COVID‐19 pandemic. The majority of the clients who were referred back were for combined phlebotomy and ART refill visits at Hangdong Hospital rather than for the ART mailing service, which could indicate a change in preference due to the COVID‐19 preventive measures. This suggested that the KP‐led service remained a viable ART refill option during the COVID‐19 pandemic. Nonetheless, the implementation context was critical to the outcomes and their interpretation. Further study should incorporate qualitative research to understand the service operation and the decision to accept and switch ART service models among the clients in the context of each site.

There are several limitations to this study. Firstly, the sample size was small and statistical significance must be interpreted with caution. The current service had rigid eligibility criteria, which might restrict the number of clients enrolled in this study. Future service should explore expanding the eligibility criteria, such as allowing people with co‐morbidities under control and/or detectable VL, to make the KP‐led service accessible to more clients. Secondly, our study population consisted mostly of MSM. More efforts are needed to reach and screen TGW with HIV, such as by integrating gender‐affirming service with HIV service, which has been done successfully for HIV testing and prevention [35, 36]. Additionally, the KP‐led model could potentially be applied to other KPs with their leadership and further service differentiation to meet the needs of specific KPs. Thirdly, the comparison group was composed of ART clients from various models offered by the hospital. However, the restriction of eligibility criteria made those who accepted and declined the KP‐led service somewhat comparable, which made outcome comparison appropriate. Lastly, this analysis extracted existing data from the CBO and hospital databases, and many important demographic, behavioural and psychosocial variables were missing or not collected, which might limit our understanding of the client characteristics for further service differentiation and should be captured in future studies.

## 5 CONCLUSIONS

Our finding reported moderate acceptability and retention in care among MSM and TGW who accepted the KP‐led HIV treatment service. Nonetheless, multiple service models should be available to provide choices and cater to the changing preferences, health needs and life situations of the diverse KPs with HIV in order to provide truly people‐centred care.

## COMPETING INTERESTS

All authors declare no competing interests related to this work.

## AUTHORS’ CONTRIBUTIONS

PL, JM, PN, SS, SS, PP, PP, RAR and NP designed and led the implementation of the key population‐led HIV treatment programme. SL and SA coordinated the programme and monitored the data. SL, PS and RR developed the analysis plan. SL and PS analysed the data. SL wrote the first draft. All authors reviewed and approved the manuscript. SL revised the manuscript according to the comments received. All authors approved the final version of the manuscript. PP and NP secured the funding.

## FUNDING

This study was supported by the US Agency for International Development (USAID) and US President's Emergency Plan for AIDS Relief (PEPFAR) through the Linkages Across the Continuum of HIV Services for Key Populations cooperative agreement (AID‐OAA‐A‐14‐0045) managed by FHI 360.

## Supporting information

Supporting information

## Data Availability

The data that support the findings of this study are available from the corresponding author upon reasonable request.
