# Supplementary material for: Acceptability and retention of the key population‐led HIV treatment service for men who have sex with men and transgender women living with HIV in Thailand
Source: J Int AIDS Soc. 2023 Feb 9;26(2):e26062. doi: 10.1002/jia2.26062 (PMC9910427; doi:10.1002/jia2.26062)
Supplement: Supplementary file 1 — Supporting information [file JIA2-26-e26062-s001.docx]

**Supporting information**

**Table S1.** Characteristics and outcomes stratified by hospitals.

|  | **Nakornping Hospital** | | | **Hangdong Hospital** | | | **Hatyai Hospital** | | |
| --- | --- | --- | --- | --- | --- | --- | --- | --- | --- |
|  | **Accepted** | **Declined** | **p-value** | **Accepted** | **Declined** | **p-value** | **Accepted** | **Declined** | **p-value** |
|  | **(N=21)** | **(N=5)** |  | **(N=13)** | **(N=2)** |  | **(N=38)** | **(N=80)** |  |
| **Age (Years)** |  |  |  |  |  |  |  |  |  |
| Median (IQR) | 26.0 (24.0-31.0) | 33.0 (23.0-34.0) | 0.601^a^ | 29.0 (24.0-31.0) | 28.5 (27.8-29.3) | 0.932^a^ | 31.0 (27.0-40.0) | 29.5 (26.0-35.0) | 0.294^a^ |
| **Age (Years)** |  |  | 0.628^b^ |  |  | 0.524^b^ |  |  | 1.000^b^ |
| <25 | 6 (29 %) | 2 (40 %) |  | 5 (38 %) | 0 (0 %) |  | 4 (11 %) | 9 (11 %) |  |
| >=25 | 15 (71 %) | 3 (60 %) |  | 8 (62 %) | 2 (100 %) |  | 34 (89 %) | 71 (89 %) |  |
| **Duration on ART (Years)** |  |  |  |  |  |  |  |  |  |
| Median (IQR) | 4.10 (3.19-4.43) | 2.78 (2.21-3.86) | 0.085^a^ | 1.65 (1.18-1.89) | 2.02 (1.81-2.24) | 0.497^a^ | 3.89 (3.14-4.58) | 3.65 (2.55-5.25) | 0.674^a^ |
| **Duration on ART (Years)** |  |  | 0.162^b^ |  |  | 0.476^b^ |  |  | 0.067^b^ |
| 1 | 1 (5 %) | 1 (20 %) |  | 10 (77 %) | 1 (50 %) |  | 2 (5 %) | 11 (14 %) |  |
| 2 | 4 (19 %) | 2 (40 %) |  | 2 (15 %) | 1 (50 %) |  | 7 (18 %) | 23 (29 %) |  |
| 3 | 4 (19 %) | 2 (40 %) |  | 1 (8 %) | 0 (0 %) |  | 11 (29 %) | 10 (12 %) |  |
| 4 | 10 (48 %) | 0 (0 %) |  | 0 (0 %) | 0 (0 %) |  | 11 (29 %) | 14 (18 %) |  |
| 5 and above | 2 (10 %) | 0 (0 %) |  | 0 (0 %) | 0 (0 %) |  | 7 (18 %) | 22 (28 %) |  |
| **Retention at month 3** |  |  | NA |  |  | 0.200^b^ |  |  | **0.010**^b^ |
| In care | 21 (100 %) | 5 (100 %) |  | 3 (23 %) | 2 (100 %) |  | 34 (89 %) | 80 (100 %) |  |
| Referred back to hospital (CBO arm only) | 0 (0 %) | NA |  | 8 (62 %) | NA |  | 3 (8 %) | NA |  |
| Transferred out to other hospital | 0 (0 %) | 0 (0 %) |  | 2 (15 %) | 0 (0 %) |  | 1 (3 %) | 0 (0 %) |  |
| LTFU | 0 (0 %) | 0 (0 %) |  | 0 (0 %) | 0 (0 %) |  | 0 (0 %) | 0 (0 %) |  |
| Death | 0 (0 %) | 0 (0 %) |  | 0 (0 %) | 0 (0 %) |  | 0 (0 %) | 0 (0 %) |  |
| **Retention at month 6** |  |  | 1.000^b^ |  |  | 0.067^b^ |  |  | **0.018**^b^ |
| In care | 19 (90 %) | 5 (100 %) |  | 2 (15 %) | 2 (100 %) |  | 31 (82 %) | 75 (94 %) |  |
| Referred back to hospital (CBO arm only) | 2 (10 %) | NA |  | 9 (69 %) | NA |  | 4 (11 %) | NA |  |
| Transferred out to other hospital | 0 (0 %) | 0 (0 %) |  | 2 (15 %) | 0 (0 %) |  | 2 (5 %) | 3 (4 %) |  |
| LTFU | 0 (0 %) | 0 (0 %) |  | 0 (0 %) | 0 (0 %) |  | 1 (3 %) | 1 (1 %) |  |
| Death | 0 (0 %) | 0 (0 %) |  | 0 (0 %) | 0 (0 %) |  | 0 (0 %) | 1 (1 %) |  |
| **Retention at month 9** |  |  | 0.298^b^ |  |  | 0.057^b^ |  |  | **<0.001**^b^ |
| In care | 15 (71 %) | 5 (100 %) |  | 1 (8 %) | 2 (100 %) |  | 27 (71 %) | 75 (94 %) |  |
| Referred back to hospital (CBO arm only) | 6 (29 %) | NA |  | 9 (69 %) | NA |  | 8 (21 %) | NA |  |
| Transferred out to other hospital | 0 (0 %) | 0 (0 %) |  | 3 (23 %) | 0 (0 %) |  | 2 (5 %) | 3 (4 %) |  |
| LTFU | 0 (0 %) | 0 (0 %) |  | 0 (0 %) | 0 (0 %) |  | 1 (3 %) | 1 (1 %) |  |
| Death | 0 (0 %) | 0 (0 %) |  | 0 (0 %) | 0 (0 %) |  | 0 (0 %) | 1 (1 %) |  |
| **Retention at month 12** |  |  | 0.632^b^ |  |  | **0.010**^b^ |  |  | **<0.001**^b^ |
| In care | 15 (71 %) | 5 (100 %) |  | 0 (0 %) | 2 (100 %) |  | 26 (68 %) | 73 (91 %) |  |
| Referred back to hospital (CBO arm only) | 5 (24 %) | NA |  | 10 (77 %) | NA |  | 8 (21 %) | NA |  |
| Transferred out to other hospital | 1 (5 %) | 0 (0 %) |  | 3 (23 %) | 0 (0 %) |  | 3 (8 %) | 4 (5 %) |  |
| LTFU | 0 (0 %) | 0 (0 %) |  | 0 (0 %) | 0 (0 %) |  | 1 (3 %) | 2 (2 %) |  |
| Death | 0 (0 %) | 0 (0 %) |  | 0 (0 %) | 0 (0 %) |  | 0 (0 %) | 1 (1 %) |  |
| **VL testing in the past 12 months** |  |  | 0.155^b^ |  |  | 1.000^b^ |  |  | **<0.001**^b^ |
| Tested | 2 (10 %) | 2 (40 %) |  | 10 (77 %) | 2 (100 %) |  | 35 (92 %) | 36 (45 %) |  |
| Not tested | 19 (90 %) | 3 (60 %) |  | 3 (23 %) | 0 (0 %) |  | 3 (8 %) | 44 (55 %) |  |
| **VL results in the past 12 months** |  |  | 0.155^b^ |  |  | 1.000^b^ |  |  | **<0.001**^b^ |
| <50 copies/mL | 2 (10 %) | 1 (20 %) |  | 10 (77 %) | 2 (100 %) |  | 35 (92 %) | 36 (45 %) |  |
| =>50 copies/mL | 0 (0 %) | 1 (20 %) |  | 0 (0 %) | 0 (0 %) |  | 0 (0 %) | 0 (0 %) |  |
| Not tested | 19 (90 %) | 3 (60 %) |  | 3 (23 %) | 0 (0 %) |  | 3 (8 %) | 44 (55 %) |  |
| ^a^Kruskal-Wallis test, ^b^Fisher's exact test | | | | | | | | | |

Interquartile range, IQR; antiretroviral therapy, ART; community-based organization, CBO; loss to follow-up, LTFU; not applicable, NA; viral load, VL; milliliter, mL.

**Table S2.** Characteristics and outcomes stratified by time of the first ART refill visit after screening (i.e. before and after median date, October 22, 2019)

|  | **Before** | | | **After** | | |
| --- | --- | --- | --- | --- | --- | --- |
|  | **Accepted** | **Declined** | **p-value** | **Accepted** | **Declined** | **p-value** |
|  | **(N=37)** | **(N=40)** |  | **(N=35)** | **(N=47)** |  |
| **Age (Years)** |  |  |  |  |  |  |
| Median (IQR) | 27.0 (25.0-31.0) | 29.5 (26.8-34.0) | 0.085^a^ | 31.0 (27.5-36.0) | 30.0 (26.0-35.0) | 0.455^a^ |
| **Age (Years)** |  |  | 0.866^b^ |  |  | 0.191^c^ |
| <25 | 8 (22 %) | 7 (18 %) |  | 7 (20 %) | 4 (9 %) |  |
| >=25 | 29 (78 %) | 33 (82 %) |  | 28 (80 %) | 43 (91 %) |  |
| **Duration on ART (Years)** |  |  |  |  |  |  |
| Median (IQR) | 3.90 (3.16-4.30) | 3.34 (2.26-5.07) | 0.571^a^ | 3.14 (1.65-4.52) | 3.64 (2.57-5.02) | 0.092^a^ |
| **Duration on ART (Years)** |  |  | **0.002**^c^ |  |  | 0.123^c^ |
| 1 | 0 (0 %) | 6 (15 %) |  | 13 (37 %) | 7 (15 %) |  |
| 2 | 9 (24 %) | 13 (32 %) |  | 4 (11 %) | 13 (28 %) |  |
| 3 | 10 (27 %) | 5 (12 %) |  | 6 (17 %) | 7 (15 %) |  |
| 4 | 15 (41 %) | 6 (15 %) |  | 6 (17 %) | 8 (17 %) |  |
| 5 and above | 3 (8 %) | 10 (25 %) |  | 6 (17 %) | 12 (26 %) |  |
| **Retention at month 3** |  |  | 0.228^c^ |  |  | **<0.001**^c^ |
| In care | 35 (95 %) | 40 (100 %) |  | 23 (66 %) | 47 (100 %) |  |
| Referred back to hospital (CBO arm only) | 2 (5 %) | NA |  | 9 (26 %) | NA |  |
| Transferred out to other hospital | 0 (0 %) | 0 (0 %) |  | 3 (9 %) | 0 (0 %) |  |
| LTFU | 0 (0 %) | 0 (0 %) |  | 0 (0 %) | 0 (0 %) |  |
| Death | 0 (0 %) | 0 (0 %) |  | 0 (0 %) | 0 (0 %) |  |
| **Retention at month 6** |  |  | 0.169^c^ |  |  | **<0.001**^c^ |
| In care | 31 (84 %) | 36 (90 %) |  | 21 (60 %) | 46 (98 %) |  |
| Referred back to hospital (CBO arm only) | 4 (11 %) | NA |  | 11 (31 %) | NA |  |
| Transferred out to other hospital | 1 (3 %) | 2 (5 %) |  | 3 (9 %) | 1 (2 %) |  |
| LTFU | 1 (3 %) | 1 (2 %) |  | 0 (0 %) | 0 (0 %) |  |
| Death | 0 (0 %) | 1 (2 %) |  | 0 (0 %) | 0 (0 %) |  |
| **Retention at month 9** |  |  | **0.005**^c^ |  |  | **<0.001**^c^ |
| In care | 27 (73 %) | 36 (90 %) |  | 16 (46 %) | 46 (98 %) |  |
| Referred back to hospital (CBO arm only) | 8 (22 %) | NA |  | 15 (43 %) | NA |  |
| Transferred out to other hospital | 1 (3 %) | 2 (5 %) |  | 4 (11 %) | 1 (2 %) |  |
| LTFU | 1 (3 %) | 1 (2 %) |  | 0 (0 %) | 0 (0 %) |  |
| Death | 0 (0 %) | 1 (2 %) |  | 0 (0 %) | 0 (0 %) |  |
| **Retention at month 12** |  |  | **0.009**^c^ |  |  | **<0.001**^c^ |
| In care | 25 (68 %) | 34 (85 %) |  | 16 (46 %) | 46 (98 %) |  |
| Referred back to hospital (CBO arm only) | 8 (22 %) | NA |  | 15 (43 %) | NA |  |
| Transferred out to other hospital | 3 (8 %) | 3 (8 %) |  | 4 (11 %) | 1 (2 %) |  |
| LTFU | 1 (3 %) | 2 (5 %) |  | 0 (0 %) | 0 (0 %) |  |
| Death | 0 (0 %) | 1 (2 %) |  | 0 (0 %) | 0 (0 %) |  |
| **VL testing in the past 12 months** |  |  | 0.583^b^ |  |  | **0.008**^b^ |
| Tested | 19 (51 %) | 17 (42 %) |  | 28 (80 %) | 23 (49 %) |  |
| Not tested | 18 (49 %) | 23 (58 %) |  | 7 (20 %) | 24 (51 %) |  |
| **VL results in the past 12 months** |  |  | 0.426^c^ |  |  | **0.006**^c^ |
| <50 copies/mL | 19 (51 %) | 16 (40 %) |  | 28 (80 %) | 23 (49 %) |  |
| =>50 copies/mL | 0 (0 %) | 1 (2 %) |  | 0 (0 %) | 0 (0 %) |  |
| Not tested | 18 (48.6%) | 23 (57.5%) |  | 7 (20.0%) | 24 (51.1%) |  |
| ^a^Kruskal-Wallis test, ^b^Chi-squared test, ^c^Fisher's exact test | | | | | | |

Interquartile range, IQR; antiretroviral therapy, ART; community-based organization, CBO; loss to follow-up, LTFU; not applicable, NA; viral load, VL; milliliter, mL.
